# Treatment of Fistulous Ulcers

**Published:** 1876-05

**Authors:** 


### TREATMENT OF FISTULOUS ULCERS.

If the sinus is complete, and in the ischiorectal fasciæ, excision is the law. The dread of pains or the inconvenience of a wound that must lie open for a week or so, to heal properly, has prevailed with many to plead for milder measures of interference. Two such have been proposed, the ligature and stimulant injections. The latter is a blind experiment, and leans for success too much on luck. Solutions of iodine and silver are thrown into the fistulæ for some time, in the belief that the pseudo-mucous lining can thus be destroyed. The injections must be followed up vigoronsly. The pain is considerable, and of a smarting character; the operation can only be ruled by a surgeon or experienced assistant. Some exception may be taken to the impropriety of this method when the fistulæ are external. Still, patients who elect this treatment, early and cheerfully abandon it for the knife.

The ligature purports to do just what we expect of the bistoury, with less hemorrhage, for more delicate patients, without anæsthetics, but does not declare for so rapid a recovery, so thorough an obliteration of the walls, and drives the subject through a series of tortures, as the cord is at intervals tightened. It is, also, to be remembered that this mode of strangulation may start a flow of pus, or an exhaustive drain of sloughing products that may in the end tally greater against the vigor of the person than the fancied shock of an incised wound. A ligature known as the "elastic" is praised by Dittel. Moliere and Allingham have not been slow in endorsing it. They argue for it that it does not incapacitate the party for work, and that the tension can be incised so artfully that the fistula will break down without pain. It has not been my fortune to try this rubber cord, and I have so mean a faith in the theory of its operation as to contract with myself never to use it. It strikes me this experiment promises a success only when the internal aperture is above the internal sphincter.

The simple noose has been allowed five trials by me. Two of the cases had been imperfectly cut by another surgeon. These five martyrs suffered the annoyance for a week, on the average, then cursed the string, and ordered me, in a few earnest idioms, to lay bare or rip up the sinuses. Setons are open to the same objection, that, instead of stimulating a new process of repair, they generally make the canals shed flakes of dead lymph. If inserted, they should be fresh every third day, and of oakum. In anal sinuses they are quite popular, and occasionally irritate the walls favorably. The "galvanic cautery" is too expensive and formidable an apparatus for general patronage. A piece of iron wire heated white-hot is just as good. So far, we have conceived and treated of fistulæ as in the territory of the anus. *Pressure* may be employed, in addition to the measures enumerated, quite advantageously, when fistulæ dissect exposed surfaces on the trunk. Compresses may be strapped on the roof of such sinuses so firmly as to coax an agglutination on both sides. Vesico-urethral and recto-vaginal fistulæ are not counted in this class, as they are lacerations of septa, and come in the province of wounds and their appropriate treatment. Ingenious as are the devices for the cure of fistulæ, they can never supplant a judicious sweep of the bistoury. Why sinuses are so stubborn in healing, when cut, is, not because the section of the muscles is too severe, or because the loss of blood compromises the life of the fibres, but through surgical blunders: (*a*) the knife is not pushed deep or high enough to extinguish the full extent of the track; (*b*) the contraction of the muscles perseveres from imperfect division; (*c*) *the cavity has not been packed properly.* In these errors lies the secret of the failure. The sinuses may have been thoroughly exposed, the surgeon considers his service done, neglects the dressing, or intrusts it to an ignorant or careless nurse. To his surprise the wound shuts up prematurely, with the canal not obliterated, and the granulations have not revived fast enough to consolidate the cavity. In operating on these ulcers, I have learned to be satisfied to carry the knife *to* the internal aperture, *then stop*, and cut out. This division concluded, I scratch the floor of the fistula with the point of the bistoury, in parallel lines, to make sure havoc of the membrane. The bleeding arrested, I stuff the wound to the edge with lint soaked in a solution of cup. sulph. (gr. ij to aq. oz. j), and adapt a pad to the parts The wad is to be drawn on the fourth day, and the wound syringed with diluted port wine. At every succeeding examination the lint should be interposed with less pressure ; but the *packing must not be discontinued until the granulations have filled the track.* The union of the cut edges may then be allowed. When the fistula is multilocular, or has several avenues winding from it into the suburbs, nothing short of a generous dissection and expose of every abscess and canal will satisfy. The question has been a good deal mooted whether fistulæ in tuberculous subjects should be closed. I am no convert to the tenet that such a sore is a counter-irritant. Experience asserts of fistulous patients that they are invariably tried and debilitated by the drain. It is to me an adulteration of good sense to countenance such a leak, when our best therapeutists insist on every measure for husbanding the strength of phthisical subjects. If counter-irritation is necessary, we can start it in more convenient and agreeable quarters than the buttock, and define it. To let such an issue stand, to ward off or postpone the liquefaction of tnbercle, is as plausible and logical as to forbear checking a chronic diarrhœa or hemorrhage for fear of some revulsion or kick elsewhere. The constitutional treatment of fistulæ is almost *nil.* The bowels are, of course, to be kept regular and free in their action Constipation is a common complaint, and is to be corrected by farinaceous food and fruit, as the pulp of prunes or figs, and gentle laxatives. If clysters are preferred, to increase the alvine urgency, warm water is sufficiently persuasive. A

course of tonics is indicated if the system is much relaxed, and pain is at once to be blunted by local or hypodermic medication with the salts of morphia. Some years since, a preparation known as "Ward's paste," a confection of black pepper, enjoyed quite a reputation as a specific for rectal ulcers. Its stimulating properties were awarded a magic energy to arouse a healthy inflammation. Time has stripped this panacea, as it has the other boons of our wiseacres in disease, of all its charms, and excision is now accepted as alone orthodox. If more scrupulous care is given to keep the walls of cut sinuses from uniting until the granulations consolidate them, fistulæ will not bother us in contracting, and crusades against the cold knife will be stilled.

